# News and Miscellany

**Published:** 1905-06

**Authors:** 


### News and Miscellany.

---

Dr. J. W. McLaughlin, Sr., Professor of Medicine, and Dr. Jno. T. Moore, Adjunct Professor of Medicine in the Medical Department of the University of Texas, have resigned their respective chairs. Their successors have not been announced.

---

San Antonio, Texas, June 8, 1905.

*Editor the Texas Medical Journal, Austin, Texas.*

Dear Sir: As Chairman of the Committee on Patent Medicines of the Fifth District Medical Association, I wish to request any physician who has seen fatal or disastrous effects following their use to send me an account of the same or any item of interest bearing upon the subject, and greatly oblige the committee.

Dr. B. F. Kingsley, Chairman.

---

Married in Austin, June 1st (inst.), Dr. Geo. R. Tabor, State Health Officer, to Miss Ann Barton, of Austin. No cards.

---

Dr. J. Hicks Florence, quarantine officer at Brownsville, is acting State Health Officer at Austin during Dr. Tabor's absence on his wedding trip.

---

The Texas State Dental Association held its twenty-fifth annual meeting at Austin May 18th, 19th and 20th. There was a large attendance, about one hundred. They accepted the State Medical Association's invitation, through committee, to affiliate with same and will send two delegates to Fort Worth meeting next April. Dr. Turner, of Belton, was elected President, and Dr. Rathbone, of Cuero, First Vice-President. Dr. Bush Jones, of Dallas, is Secretary.

For Sale.—$1500 to $2000 practice to purchaser of my residence property at Turlington, a post village in Freestone county, Texas. Address, Dr. M. M. Turlington, Turlington, Texas.

